# Sequential analysis of single-center experience of living donor kidney transplants with several vascular anastomosis techniques

**DOI:** 10.3906/sag-2007-285

**Published:** 2021-06-28

**Authors:** Berrin Papila KUNDAKTEPE, Ali Vedat DURGUN, Ertuğrul GÖKSOY, Salih PEKMEZCİ, Metin KAPAN, Kaya SARIBEYOĞLU, Mehmet VELİDEDEOĞLU, Mehmet ELİÇEVİK

**Affiliations:** 1 Department of General Surgery, Cerrahpaşa Faculty of Medicine, İstanbul University-Cerrahpaşa, İstanbul Turkey; 2 Department of General Surgery, Faculty of Medicine, İstinye University Liv Hospital, İstanbul Turkey; 3 Department of General Surgery, Carl Thiem Klinikum, Cottbus Germany; 4 Department of Pediatric Surgery, Cerrahpaşa Faculty of Medicine, İstanbul University-Cerrahpasa, İstanbul Turkey

**Keywords:** Renal transplantation, graft vascular variation, anastomosis, complication

## Abstract

**Background and aim:**

Vascular variations of grafts are handled with various reconstruction techniques in renal transplantation. We aimed to analyze the effects of these reconstruction techniques and sites on patient/graft outcomes.

**Materials and methods:**

Renal transplantation cases at the Transplantation Unit of the General Surgery Department, İstanbul University Cerrahpaşa Medical Faculty between January 1st, 2000 and December 31st, 2012 were analyzed retrospectively. Postoperative duplex ultrasound results, urea-creatinine reduction rates, and complications were evaluated.

**Results:**

There were 228 living-donor transplantation cases evaluated. For single-renal-artery living-donor transplantations, there were 45 end-to-side external iliac artery, 15 end-to-side internal iliac artery, 152 end-to-end internal iliac artery, and 3 end-to-side common iliac artery anastomoses performed. In cases with double-arteries, 3 had end-to-side external iliac artery anastomoses, and 10 had endto-end internal iliac artery anastomoses. No statistically significant differences were found between reconstruction techniques with regard to complications or urea-creatinine reduction rates.

**Conclusion:**

Internal, external, and common iliac arteries can be safely used for anastomoses. The presence of more than one renal artery creates no short or long-term problems when a side-to-side anastomosis is initially performed.

## 1. Introduction

Kidney transplantation has been a life-saving solution for patients in renal failure. The most common cause of chronic renal failure is diabetes mellitus. The other frequently met indications for renal transplantation are hypertension, glomerulonephritis, and cystic kidney disease [1]. With a history of more than half a century, this procedure has been improved over time. Due to advances in the design of surgical instruments, immunosuppressive and antibiotic agents, and measures to prevent ischemia of grafted kidneys, the outcomes of renal transplantation procedures have improved in recent years.

The principles of the technique for vascular anastomosis described by Carrel in 1902 and the technique for implantation to the iliac vessels described by Kuss in 1951 are currently in use [2,3]. Selection of a kidney appropriate for transplantation involves various factors including the size and functional status of the organ, presence of anatomical variations, the status and number of supplying arteries, and the venous drainage [4]. A donor kidney is placed to the right or left iliac fossa, with vascularization driven from the iliac vessels. The right side is usually preferred as the initial transplantation site because the iliac vessels in this area follow a more superficial course compared to the left side. The left side might be preferred when the recipient is also a candidate for pancreas transplantation, when previous renal transplantation has been performed on the right side, or in the presence of right-sided vascular pathology. When multiple arteries are present, the procedure requires either the reduction of the number of arteries to one or performing in situ multiple anastomoses. Another option is using a Carrel patch if it is not a living-donor transplantation [5]. 

Complications may be seen due to restoration procedures by surgery or percutaneous stenting. Renal artery stenosis is a common and challenging complication [6]. In the presence of a single artery, the rate of arterial stenosis is 8% when an end-to-end anastomosis is performed between the internal iliac artery and the renal artery [7]. Orlic et al. reported that they had reduced the rate of stenosis to 0.72% by performing an end-to-side anastomosis to either the external or common iliac artery [7]. 

In this study, we aimed to analyze and compare the effects of different techniques and sites of anastomosis on the outcomes of the patients and the grafts together with complications that may have occurred in the early and late postoperative periods.

## 2. Materials and methods

This retrospective study was conducted in the Transplantation Unit of the Department of General Surgery at İstanbul University Cerrahpaşa Faculty of Medicine after obtaining the approval of the Ethics Board of İstanbul University Cerrahpaşa Faculty of Medicine (3707/06.02.2014). 

Records of adult transplantation patients who had surgery in the Transplantation Unit of the Department of General Surgery in İstanbulUniversity Cerrahpaşa Faculty of Medicine between January 1st, 2000 and December 31st, 2012 were reviewed. An Abdominal CT-angiography protocol was applied in the selection of donor cases and Doppler ultrasonography was used for follow-up of the recipients. 

The status of the renal arteries of the donors, the types and sites of anastomosis, the occurrence of rejection or complications in the early and late postoperative periods, the postoperative renal arterial flow rate, and the resistive index obtained by Doppler ultrasonography were recorded on the study data sheet together with the levels of urea and creatinine on the postoperative 1st and 7th days, 1st and 6th months, and at the 1st year follow-up. 

### 2.1. Statistical analysis

The continuous variables are presented as mean+/-SD or median (min-max) according to data distribution normality and categorical variables are presented as n (%).  NCSS (Number Cruncher Statistical System) Kaysville, Utah, USA) program was used to perform statistical analysis. The suitability of the quantitative data to normal distribution was tested by Kolmogorov–Smirnov, Shapiro–Wilk test and graphical evaluations. Student t test was used for two-group comparisons of quantitative data with normal distribution, and Mann–Whitney U test was used for two-group comparisons of data not showing normal distribution. Kruskal–Wallis test and Bonferroni Dunn test were used for paired comparisons of three and more groups that did not show normal distribution. In comparison of qualitative data, Pearson chi-square test, Fisher-Freeman-Halton exact test and Fisher’s exact test were used. P-value of <0.05 was accepted as significant.

## 3. Results

Records of adult transplantation patients who had been operated in the Transplantation Unit of the Department of General Surgery at İstanbul University Cerrahpaşa Faculty of Medicine between January 1st, 2000 and December 31st, 2012 were reviewed. A total of 228 patients, 155 (68.0%) male and 73 (32.0%) female, were found to have received their grafts from living donors. The mean age of the recipients was 38.45 years. The living donors were 96 (42.1%) mothers, 49 (21.5%) fathers, 39 (17.1%) siblings, 36 (15.8%) spouses, 6 (2.6%) second-degree relatives, and 2 (0.9%) nonblood relatives of the patients (Table 1). Among all patients who had undergone renal transplantation, the etiology was unidentified in 80 (35.1%). Of the remaining patients, renal transplantation was performed due to chronic glomerulonephritis (including focal segmental glomerulonephritis, membranoproliferative glomerulonephritis, diffuse mesangial proliferative glomerulonephritis) in 45 (19.7%), vesicoureteral reflux in 31 (13.6%), hypertension in 16 (7%), amyloidosis in 8 (3.5%), IgA nephropathy in 8 (3.5%), nephrolithiasis in 7 (3.1%), diabetes mellitus in 7 (3.1%), pyelonephritis in 7 (3.1%), ureteropelvic junction obstruction in 4 (1.75%), gout in 1 (0.4%), and other reasons (Alport syndrome, Henoch-Schonlein vasculitis, and pre-eclampsia) in 14 (6.1%) cases. 

**Table 1:** Evaluation of anastomosis types according to demographic features.

		Anastomosis type						p
		1External iliac artery end-to-side (n = 45)	2Internal iliacartery end-to-side (n = 152)	•Internal iliac artery end-to-end (n = 3)	•Common iliac artery end-to-side (n = 3)	3External iliac artery end-to-side, two arteries(n = 15)	4Internal iliacartery end-to-end, two arteries(n = 10)	
Age (year)	Q1 - Q3 (Median)	28.5 - 50.5 (40)	31 - 42 (35)	39 - 54 (54)	57 - 60 (58)	32 - 50 (37)	28.5 - 39.8 (34.5)	a0.269
	Mean ± SD	40.64 ± 12.69	37.17 ± 9.82	49.00 ± 8.66	58.33 ± 1.53	40.73 ± 11.40	35.50 ± 8.76	
Gender; n (%)	Female	15 (33.3)	48 (31.6)	0 (0)	1 (33.3)	5 (33.3)	4 (40.0)	c0.938
	Male	30 (66.7)	104 (68.4)	3 (100)	2 (66.7)	10 (66.7)	6 (60.0)	
Donors; n (%)	Mothers	17 (37.8)	68 (44.7)	0 (0)	0 (0)	7 (46.7)	4 (40.0)	d0.849
	Fathers	6 (13.3)	36 (23.7)	0 (0)	0 (0)	3 (20.0)	4 (40.0)	c0.235
	Siblings	9 (20)	28 (18.4)	0 (0)	0 (0)	1 (6.7)	1 (10.0)	c0.723
	Spouses	10 (22.2)	16 (10.5)	3 (100)	3 (100)	4 (26.7)	0 (0)	c0.048*
	Nonblood relativesof the patients	1 (2.2)	1 (0.7)	0 (0)	0 (0)	0 (0)	0 (0)	c0.531
	Second-degreerelatives	2 (4.4)	3 (2.0)	0 (0)	0 (0)	0 (0)	1 (10.0)	c0.217
Early complications;n (%)	Complications ( - )	34 (75.6)	113 (74.3)	2 (66.7)	3 (100)	10 (66.7)	7 (70.0)	c0.855
	Complications ( + )	11 (24.4)	39 (25.7)	1 (33.3)	0 (0)	5 (33.3)	3 (30.0)	
Late complications;n (%)	Complications ( - )	33 (73.3)	122 (80.3)	2 (66.7)	2 (66.7)	10 (66.7)	5 (50.0)	c0.095
	Complications ( + )	12 (26.7)	30 (19.7)	1 (33.3)	1 (33.3)	5 (33.3)	5 (50.0)	

aKruskal–Wallis test cFisher–Freeman–Halton–Exact test dPearson Ki-kare test

The external, internal, and common iliac arteries were used for anastomoses in the transplantation of 215 living-donor with single-renal-artery kidneys. In single-renal-artery living-donor transplantations, anastomoses of end-to-side external iliac artery, end-to-side internal iliac artery, end-to-end internal iliac artery, and end-to-side common iliac artery were performed in 45, 15, 152, and 3 cases, respectively (Figure 1a, 1b, 1c). In cases with double-arteries, following side-to-side anastomosis of renal arteries (double-barrel technique), three had end-to-side external iliac artery anastomoses, and ten had end-to-end internal iliac artery anastomoses. 

**Figure 1a:**
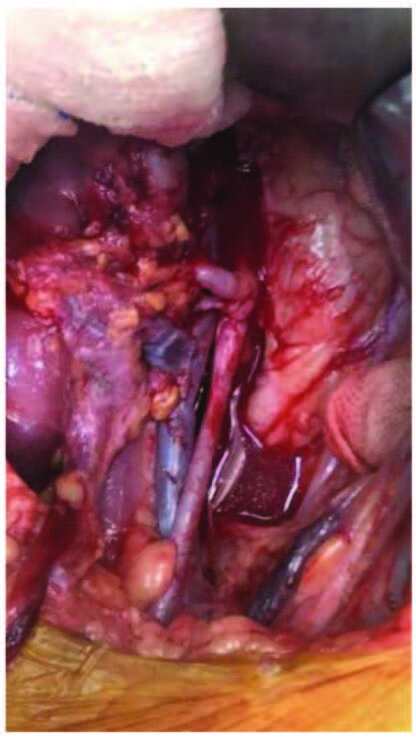
Double artery anastomosis.

**Figure 1b:**
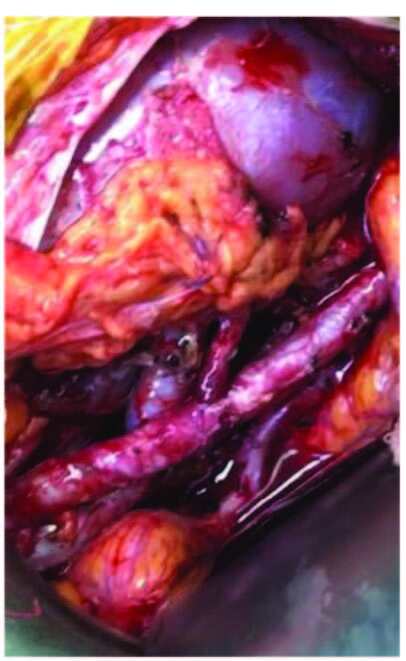
End-to-side external iliac artery anastomosis.

**Figure 1c:**
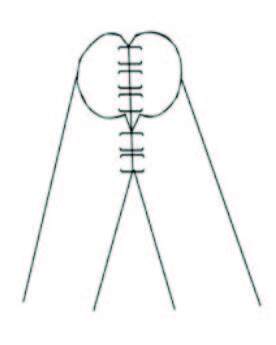
Double barrel technique.

The number of anastomoses using the common iliac artery was small; therefore, we considered the common iliac and external iliac arteries as a single group compared to the largest group involving the internal iliac artery as the anastomotic site, but not considering the arterial status of the graft. We found significant differences regarding day-1 creatinine level (p = 0.048), and day-7 creatinine level (p = 0.001) between the external/common and the internal iliac artery groups with living-donor transplants. There was no significant difference regarding the other parameters examined (Table 2). 

**Table 2:** The comparison of the anastomosis sites in the living-donor group (external/common iliac artery and internal iliac artery) without taking the graft renal arterial status into consideration.

			Anastomosis type		gp
		Total (n = 228)	External or Common iliac artery (n = 51)	Internal iliac artery(n = 177)	
Day 1 urea (mg/dL)	Q1-Q3 (Median)	51–93.8 (66)	49–94 (68)	51–93.5 (64)	0.951
	Mean ± SD	74.62 ± 33.90	74.14 ± 33.06	74.76 ± 34.23	
Day 7 urea (mg/dL)	Q1 - Q3 (Median)	46.3–96.8 (68)	42–91 (60)	47.5–101.5 (70)	0.056
	Mean ± SD	84.09 ± 57.02	69.43 ± 40.8	88.32 ± 60.34	
Day 30 urea (mg/dL)	Q1 - Q3 (Median)	39–63 (50)	38–63 (50)	40v 63 (50)	0.751
	Mean ± SD	55.36 ± 26,74	54.69 ± 26.57	55.56 ± 26.87	
Month 6 urea (mg/dL)	Q1 - Q3 (Median)	31–46.5 (37)	32–49 (41)	31–46 (36)	0.098
	Mean ± SD	39.68 ± 13.25	41.43 ± 12.92	39.17 ± 13.34	
Year 1 urea (mg/dL)	Q1 - Q3 (Median)	30v 45 (38)	32–48 (40)	29–45 (37)	0.162
	Mean ± SD	39.49 ± 13.84	41.14 ± 13.47	39.01 ± 13.95	
Day 1 creatinine(mg/dL)	Q1 - Q3 (Median)	2.4–4.9 (3,3)	2–4 (3.2)	2.4–5.1 (3.4)	0.048*
	Mean ± SD	3.97 ± 2.34	3.28 ± 1.52	4.16 ± 2.50	
Day 7 creatinine(mg/dL)	Q1 - Q3 (Median)	1.2–2.3 (1.5)	1.1–1.6 (1.3)	1.3–2.6 (1.7)	0.001**
	Mean ± SD	2.36 ± 2.27	1.63 ± 1.14	2.57 ± 2.47	
Day 30 creatinine(mg/dL)	Q1 - Q3 (Median)	1–1.6 (1.3)	0.9–1.4 (1.2)	1–1.6 (1.3)	0.071
	Mean ± SD	1.42 ± 0.99	1.24 ± 0.34	1.47 ± 1.10	
Month 6 creatinine(mg/dL)	Q1 - Q3 (Median)	1.1–1.5 (1.3)	1–1.4 (1.3)	1.1–1.5 (1.3)	0.347
	Mean ± SD	1.31 ± 0.35	1.24 ± 0.30	1.33 ± 0.37	
Year 1 creatinine(mg/dL)	Q1 - Q3 (Median)	1.1–1.5 (1.3)	1–1.4 (1.2)	1.1–1.5 (1.3)	0.057
	Mean ± SD	1.31 ± 0.45	1.20 ± 0.34	1.34 ± 0.47	
Renal artery blood flow velocity (cm/sec)	Q1 - Q3 (Median)	115–196.6 (135.3)	113.5–233 (140)	115–186.3 (133.5)	0.722
	Mean ± SD	172.88 ± 95.47	178.56 ± 98.73	170.92 ± 94.68	
Resistive Index	Q1 - Q3 (Median)	0.6–0.7 (0.6)	0.6–0.7 (0.6)	0.6–0.7 (0.6)	0.765
	Mean ± SD	0.64 ± 0.1	0.64 ± 0.12	0.64 ± 0.09	

gMann–Whitney U Test

We then searched for individualized differences between the anastomotic sites, considering the type of anastomosis, and the arterial status of the graft separately. We determined highly significant differences among the anastomotic sites with regard to day-1 urea level (p = 0.041), day-1 creatinine level (p = 0.002), day-7 urea level (p = 0.017) and day-7 creatinine level (p = 0.001) (Table 3). 

**Table 3:** The comparison of the anastomosis sites in the living-donor group, taking into consideration the type of the anastomosis and the arterial status of the graft separately. *Statistically significant.

		Anastomosis type						ap	Post Hoc;bp
		1External iliac artery end-to-side (n = 45)	2Internal iliac artery end-to-side (n = 152)	•Internal iliac artery end-to-end (n = 3)	•Common iliac artery end-to-side (n = 3)	3External iliac artery end-to-side, two arteries(n = 15)	4Internal iliac artery end-to-end, two arteries(n = 10)		
Day 1 urea(mg/dL)	Q1 - Q3 (Median)	47–92 (67)	50.3–92 (63)	80–142 (109)	62–161 (88)	46–90 (67)	79.3–135.5 (94)	0.041*	4 > 1. 2
	Mean ± SD	69.76 ± 30.17	73.27 ± 33.86	110.33 ± 31.02	103.67 ± 51.33	68.93 ± 24.45	106.20 ± 39.35		
Day 7 urea(mg/dL)	Q1 - Q3 (Median)	41–75.5 (57)	47– 97.5 (69)	47–120 (55)	111–254 (125)	52–81 (66)	54.8–246.8 (165)	0.017*	4 > 1
	Mean ± SD	62.87 ± 29.82	85.33 ± 55.42	74.00 ± 40.04	163.33 ± 78.83	71.47 ± 38.74	159.00 ± 105.67		
Day 30 urea(mg/dL)	Q1 - Q3 (Median)	33.5–62.5 (46)	40–61.8 (50)	60–120 (62)	59–103 (61)	39–76 (57)	42–77.5 (49)	0.545	-
	Mean ± SD	51.64 ± 25.29	54.83 ± 26.29	80.67 ± 34.08	74.33 ± 24.85	62.27 ± 35.57	56.67 ± 20.86		
Month 6 (mg/dL)	Q1 - Q3 (Median)	1–1.4 (1.3)	1.1–1.4 (1.3)	1.3–1.5 (1.3)	0.7–1.5 (1.3)	1.2–1.6 (1.4)	1.1–1.5 (1.3)	0.209	-
	Mean ± SD	1.24 ± 0.31	1.32 ± 0.37	1.33 ± 0.12	1.18 ± 0.39	1.43 ± 0.38	1.30 ± 0.37		
Year 1 urea(mg/dL)	Q1 - Q3 (Median)	1–1.4 (1,1)	1.1–1.5 (1.3)	1.2–1.9 (1.5)	0.8–1.3 (1.2)	1.2–1.5 (1.3)	1–1.6 (1,3)	0.414	-
	Mean ± SD	1.19 ± 0.33	1.34 ± 0.49	1.53 ± 0.32	1.08 ± 0.29	1.35 ± 0.34	1.25 ± 0.34		
Day 1 creatinine(mg/dL)	Q1 - Q3 (Median)	1.9–3.8 (2.8)	2.4–4.9 (3.3)	4–7.1 (6.9)	3.3–5.2 (3.5)	2.4–7.3 (3.1)	4.4–7.8 (5.5)	0.002**	4 > 1. 2
	Mean ± SD	3.05 ± 1.36	4.01 ± 2.40	6.02 ± 1.73	3.98 ± 1.06	4.58 ± 3.23	5.93 ± 2.27		
Day 7 creatinine(mg/dL)	Q1 - Q3 (Median)	1–1.6 (1.3)	1.2–2.5 (1.6)	1.1–6.4 (1.3)	1.4 - 3.3 (2.4)	1.3 - 1.8 (1.5)	1.4 - 9.8 (5)	0.001**	4 > 1. 2
	Mean ± SD	1.50 ± 0.92	2.40 ± 2.09	2.92 ± 3.02	2.37 ± 0.95	2.38 ± 3.17	5.57 ± 4.40		
Day 30 creatinine(mg/dL)	Q1 - Q3 (Median)	0.9 - 1.5 (1.2)	1 - 1.6 (1.3)	1.1 - 2.3 (1.2)	1.1–1.4 (1.1)	1.1–1.8 (1.5)	1–1.6 (1.4)	0.101	-
	Mean ± SD	1.23 ± 0.32	1.40 ± 0.65	1.53 ± 0.66	1.17 ± 0.17	2.27 ± 3.13	1.23 ± 0.46		
Month 6 creatinine(mg/dL)	Q1 - Q3 (Median)	32–47.5 (41)	30–45 (36)	36–63 (50)	31–50 (43)	31.8–47.3 (40)	36–53 (44)	0.446	-
	Mean ± SD	40.89 ± 13.13	38.72 ± 13.04	49.67 ± 13.5	41.33 ± 9.61	39.93 ± 8.78	45.67 ± 21.9		
Year 1 creatinine(mg/dL)	Q1 - Q3 (Median)	30.5–47 (40)	30–45 (37)	36–75 (56)	34–70 (42)	27.5–40 (34.5)	30–51 (45)	0.201	-
	Mean ± SD	39.67 ± 12.33	39.23 ± 14.32	55.67 ± 19.50	48.67 ± 18.90	35.43 ± 10.13	40.78 ± 13.09		
Renal artery blood flow velocity(cm/sec)	Q1 - Q3 (Median)	114.5–219 (140)	115–186.3 (130.5)	80–290 (130)	66–291.5 (190)	116.5–322 (158.5)	94.4–160 (137.5)	0.446	-
	Mean ± SD	179.26 ± 100.01	166.84 ± 90.79	166.67 ± 109.7	182.50 ± 112.94	216.25 ± 124.30	130.42 ± 36.69		
Resistive Index	Q1 - Q3 (Median)	0.5–0.7 (0.6)	0.6–0.7 (0.6)	0.6–1 (0.6)	0.7–0.8 (0.7)	0.6–0.8 (0.6)	0.6–0.7 (0.6)	0.172	-
	Mean ± SD	0.62 ± 0.11	0.63 ± 0.09	0.73 ± 0.24	0.75 ± 0.09	0.70 ± 0.12	0.65 ± 0.09		

aKruskal–Wallis TestbBonferroni–Dunn Test

**Figure 2:**
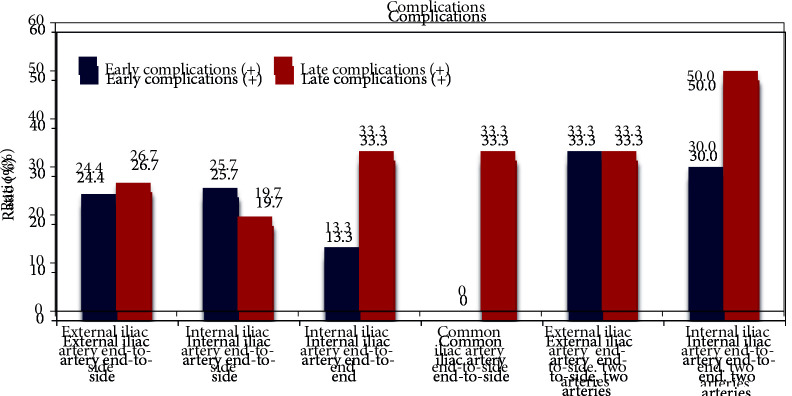
Distribution of early and late complications according to anastomosis types.

The complications that occurred in our case series included acute tubular necrosis, urinary tract dilation, lymphocele, thrombus, rejection, seroma, perirenal hematoma, urinoma, ureteral leak, stenosis, urinary tract infection and abscess, disease recurrence, ileus, renal artery stenosis, development of malignancy, drug toxicity, and nephrolithiasis. These complications were classified as early (occurring within 30 days of transplantation) and late. Acute tubular necrosis, thrombus formation, ureteral stenosis, and renal artery stenosis are important complications of vascular reconstruction and anastomosis. Acute tubular necrosis was seen in two cases in the external iliac artery end-to-side anastomosis group and in ten cases in the internal iliac artery end-to-end anastomosis group. Ureteral stenosis was seen in nine cases in the internal iliac artery end-to-end anastomosis group. Thrombus formation was seen in two cases in the internal iliac artery end-to-end anastomosis group and in one case in the internal iliac artery end-to-side anastomosis group. Renal artery stenosis was seen in five cases in the internal iliac artery end-to-end anastomosis group. In the multiple artery group, three cases of ureteral stenosis occurred in the internal iliac artery end-to-end anastomosis group. Rejection occurred in one case in the external iliac artery end-to-side anastomosis group with multiple arteries. There were no significant differences among the anastomotic sites with regard to the rates of early and late complications when the type of the anastomosis and the arterial status of the graft were considered separately (Table 1, Figure 2). Since they do not differ significantly between subgroups, the diversity and distribution of complications are not specified separately. There was no significant distribution of complications among the groups according to the anastomotic technique used. Graft loss occurred in six cases in the external iliac artery end-to-side anastomosis group, 16 cases in the internal iliac artery end-to-end anastomosis group, three cases in the internal iliac artery end-to-side anastomosis group, and one case in the common iliac artery end-to-side anastomosis group. There was no significant difference between the groups in this regard.

## 4. Discussion

In our series, chronic glomerulonephritis was the most common diagnosis among all identified cases, at 19.8%. The anastomoses were performed using the external, internal, and common iliac arteries, although they were not distributed homogeneously [8,9]. An end-to-side anastomosis was preferred for the external iliac artery, whereas both end-to-end and end-to-side anastomoses were performed on the internal iliac artery [10,11]. An end-to-end anastomosis to the internal iliac artery is preferred if the renal artery is too short or the anastomosis to the external iliac artery will be stretched or if the external iliac artery is atherosclerotic. In the patients having two arteries, these arteries were anastomosed to each other first with the fish-mouth technique or double-barrel technique then anastomosed to either the internal or external iliac artery. Highly significant differences were found between living-donor transplantation patient vessel groups with regard to 1st-day creatinine and urea, 7th-day creatinine and urea. There were no differences in the occurrence of complications and no differences were observed in the urea and creatinine levels at six months and one year. 

The frequency of having multiple renal arteries has been reported to range from 18% to 43% in a large autopsy series [12]. In our series, the total number of donor grafts with more than one renal artery was 20 (7.5%), which was lower than that reported in the large autopsy series. 

The use of donor kidneys with more than one artery has been a contentious issue due to technical difficulties, prolonged duration of warm ischemia, and a higher risk of complications such as acute tubular necrosis, potential vascular and urological complications, and even eventual loss of the allograft. In our series, ureteral stenosis was significantly higher in the internal iliac artery end-to-end anastomosis group with multiple arteries. Wolters et al. reported in 2001 that, in the presence of multiple arteries, ureteral necrosis was commonly seen due to the deterioration of perfusion caused by the ligation of the polar artery [13]. Therefore, the presence of multiple renal arteries had been considered an obstacle for performing transplantations. 

Various reports in the literature have concluded that the presence of more than one artery leads to minor consequences, not long-term derangements. Tyson et al., in their retrospective study of 584 patients, reported that the presence of multiple renal arteries did not influence the long-term survival of the allografts and the patients, although it did cause retardation of graft function [14]. A study conducted by Kok et al. [15] also demonstrated that multiple arterial and vascular reconstructions did not affect the level of creatinine in the recipient; however, they reported that the accessory artery supplying the inferior pole of the kidney was associated with increased rates of ureteric complications. Benedetti et al., in their study conducted between the years 1985 and 1993 comparing 163 patients having allografts with multiple arteries to 835 patients with single artery allografts, determined no differences regarding the development of hypertension, acute tubular necrosis, acute rejection, creatinine level, or the rates of early vascular and urologic complications in the post-transplantation period [12]. Several other studies comparing transplantations from donors with a single artery to donors with more than one artery have found similar rates of survival and similar outcomes associated with the grafts. Vasquez et al. did not report any differences between single artery and multiple artery groups regarding renal functions (serum creatinine and GFR), and the results of renal biopsies performed a year later [16,17]. Ghazanfar et al., in their study conducted in 2010, concluded that donor kidneys with multiple arteries could be used safely for renal transplantations [18].

Several studies in the literature have compared the effects of anastomotic sites and reconstruction techniques in addition to the number of renal arteries. They have reported that the presence of multiple arteries (single anastomosis, multiple anastomoses, and ligation), the type of anastomosis (end-to-end or end-to-side), and the site of anastomosis have not affected short and long-term graft outcomes. It has been demonstrated that, in the presence of multiple arteries, the end-to-end anastomosis of the main artery to the internal iliac artery or its end-to-side anastomosis to the external iliac artery neither affects the graft or patient survival nor increases the rates of vascular and urologic complications [19–21]. 

No significant difference was found compared to the literature in our study evaluating long-term complication rates and urea-creatinine levels related to the number of renal graft arteries, together with the sites and types of anastomosis in patients from various age groups with various etiologic factors who had undergone living-donor renal transplantation procedures. Thus, the presence of more than one artery should not be considered an obstacle to transplantation. We also conclude that choosing the site and type of anastomosis should not create any hesitation for long-term graft and patient outcomes.

We suggest that since the number of donors is currently limited throughout the world, donor grafts with duplicate arteries should not be excluded from the donor pool. We also suggest that further prospective studies with more extensive or multi-center series and an increased number of variations in anastomotic techniques should be conducted to reach definitive conclusions on this subject.

### 4.1. Limitations of our study

Our study had several limitations. The first limitation was its retrospective feature. Only the data present in the patient charts were recorded and analyzed leading to an inability to evaluate parameters such as the total duration of ischemia and the requirement for antihypertensive medications. Also, the risk of sexual impotence, which can occur in patients with an internal iliac anastomosis, was not questioned.

## Informed consent

The study has been approved by İstanbul University Cerrahpaşa Faculty of Medicine Ethics Board with number: 3707/06.02.2014.

## References

[ref1] (2014). Patient selection and indications for kidney transplantation. Textbook of Organ Transplantation. West Sussex.

[ref2] (2009). Kidney transplant anastomosis: internal or external iliac artery?. Urology Journal.

[ref3] (2002). Fifty years of retroperitoneal placement of renal transplants. Transplantation Proceedings.

[ref4] (2009). Maximizing the donor pool: use of right kidneys and kidneys with multiple arteries for live donor transplantation. Surgical Endoscopy.

[ref5] (2005). Management and outcome of living kidney grafts with multiple arteries. Surgery Today.

[ref6] (1993). Renal artery stenosis after renal transplantation: the impact of the hypogastric artery anastomosis. The Journal of Urology.

[ref7] (2003). Vascular complications after 725 kidney transplantations during 3 decades. Transplantation Proceedings.

[ref8] (2017). Vascular complications after renal transplant: a single-center experience. Experimental and Clinical Transplantation.

[ref9] Vice-chair) et al.

[ref10] (2012). Use of internal iliac artery as a side-to-end anastomosis in renal transplantation. Annals of the Royal College of Surgeons of England.

[ref11] (2017). Analysis of outcome of end-to-end and end-to-side internal iliac artery anastomosis in renal transplantation: Our initial experience with a case series. Urology Annals.

[ref12] (1995). Short- and long-term outcomes of kidney transplants with multiple renal arteries. Annals of Surgery.

[ref13] (2001). The anastomosis between renal polar arteries and arteria epigastrica inferior in kidney transplantation: an option to decrease the risk of ureter necrosis?. Transplant International.

[ref14] (2011). Living donor kidney transplantation with multiple renal arteries in the laparoscopic era. Urology.

[ref15] (2008). Complex vascular anatomy in live kidney donation: imaging and consequences for clinical outcome. Transplantation.

[ref16] (2010). Renal grafts with multiple arteries: a relative contraindication for a renal transplant?. Transplantation Proceedings.

[ref17] (2007). Outcome of renal allografts with multiple arteries. Transplantation Proceedings.

[ref18] (2010). The outcomes of living donor renal transplants with multiple renal arteries: a large cohort study with a mean follow-up period of 10 years. Transplantation Proceedings.

[ref19] (2013). Complications and graft survival in kidney transplants with vascular variants: our experience and literature review. Transplantation Proceedings.

[ref20] (2009). Kidney transplantation at Annunziata Hospital of Cosenza: report of 10 years experience. Transplantation Proceedings.

[ref21] (2010). The long-term outcomes of transplantation of kidneys with multiple renal arteries. Transplantation Proceedings.

